# Harnessing the Structural Variability of Doxycycline Hyclate: Mechanistic Insights and Comparative Assessment of Four Chemically Diverse Spectrophotometric Pathways

**DOI:** 10.1155/jamc/2780011

**Published:** 2026-08-30

**Authors:** Mona A. Abdel Rahman, Reem H. Obaydo

**Affiliations:** ^1^ Analytical Chemistry Department, Faculty of Pharmacy, October 6 University, P. O. Box 12858, 6 October City, Giza, Egypt, o6u.edu.eg; ^2^ Department of Analytical and Food Chemistry, Faculty of Pharmacy, Ebla Private University, Idlib, Syria

**Keywords:** doxycycline hyclate, Fast Red B, ion-pair extraction, N-bromosuccinimide, Turnbull’s blue

## Abstract

Four distinct, chemically asynchronous spectrophotometric pathways each tapping into a different reactive node of the polyfunctional doxycycline hyclate (DOX) framework were planned to bypass the limitations of single mechanism assays. It works amazingly well. By exploiting the drug’s inherent structural mutability, we developed a multipronged quantification matrix. Take the drug’s reducing capacity, for instance. In Method A, this specific driving force reduces Fe (III) in the presence of potassium ferricyanide, yielding a highly conjugated Turnbull’s blue complex characterized by a distinct bathochromic shift (*λ*_max_ 783.5 nm). Method B switches components entirely. It forces an alkaline electrophilic coupling with Fast Red B salt to lock down a resilient red azo chromophore (*λ*_max_ 498 nm). Method C determines for selective oxidation using N‐bromosuccinimide (NBS) in a strongly acidic environment to generate a stable yellow derivative (*λ*_max_ 383.5 nm) (a sequence highly dependent on strict kinetic control). Method D abandons covalent modification altogether, relying instead on the generation of a lipophilic ion‐pair associate with bromothymol blue (BTB) at pH 3.0, which partition readily into chloroform (*λ*_max_ 409.5 nm). Linearity spans diverse concentration intervals: 0.5–4, 5–60, 20–140, and 5–35 μg mL^−1^ for protocols A through D. Sensitivity peaks at a remarkably low LOD of 0.16 μg mL^−1^. Rigorous compliance with ICH validation protocols confirmed tight fidelity regarding accuracy and reproducibility. When applied to real‐world commercial capsules, the matrix remained entirely unaffected by matrix excipients. This makes it an incredibly cheap, solvent‐lean alternative for high‐throughput benchtop quality control.

## 1. Introduction

**FIGURE 1:**
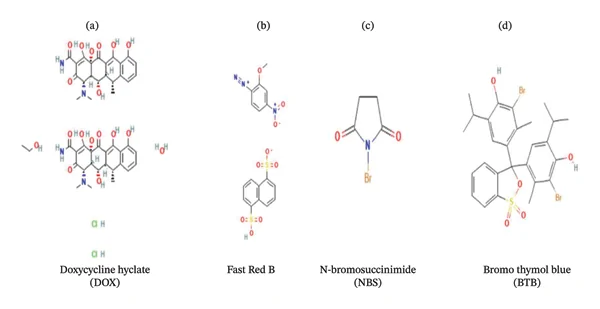
Structure of (a) doxycycline hyclate, (b) Fast Red B, (c) N‐bromosuccinimide, and (d) bromo thymol blue.

Doxycycline hyclate (DOX) is a prominent semi‐synthetic tetracycline antibiotic renowned for its broad‐spectrum efficacy against a diverse array of Gram‐positive and Gram‐negative bacteria [1]. These physicochemical properties explain its therapeutic efficacy and substantiate its clinical application in infections, including Lyme disease, anthrax, chlamydial infections, and mycoplasmal pneumonia [2]. Structurally, DOX is distinguished from other tetracyclines by the absence of a hydroxyl group at the C‐6 position, a modification that significantly enhances its lipophilicity and metabolic stability. This structural refinement facilitates superior tissue penetration and a prolonged systemic half‐life, which are vital for its clinical effectiveness [3]. Beyond its primary antimicrobial role, DOX exhibits significant immunomodulatory properties and acts as a potent inhibitor of matrix metalloproteinases (MMPs), particularly MMP‐9. These nonantibiotic properties have expanded its application to the treatment of chronic inflammatory conditions, including rheumatoid arthritis, acne vulgaris, and lupus vasculitis [4, 5]. The use of DOX in human and veterinary medicine raises concerns regarding environmental pollution and food safety due to its residues in consumable tissues, which may raise health risks and contribute to antibiotic resistance [6, 7]. DOX is chemically named hydrochloride hemiethanol hemihydrate of (4S,4aR,5S,5aR,6R,12aS)‐4‐(dimethylamino)‐3,5,10, 12,12a‐pentahydroxy‐6‐methyl‐1,11‐dioxo‐1,4,4a,5, 5a,6,11,12a‐octa‐hydrotetracene‐2‐carboxamide (Figure 1a). The complex polyhydroxylated hydronaphthacene structure of DOX, characterized by its rich electron‐donating dimethylamino and carboxamide groups, provides multiple reactive sites that facilitate a multimechanistic approach to its quantification through various analytical methods [8]. Specifically, the phenolic and enolic hydroxyl groups serve as active sites for oxidative coupling and diazotization, while the *β*‐diketone moiety facilitates stable metal‐ion chelation with multivalent cations such as Fe^3+^, Al^3+^, or Cu^2+^. Furthermore, the dimethylamino group at the C‐4 position renders the molecule an efficient n‐electron donor, enabling the formation of charge‐transfer complexes with various *π* acceptors [9].

The literature provides several analytical methods for the spectrophotometric determination of DOX [10–16], high‐performance liquid chromatography (HPLC) and ultra‐high‐performance liquid chromatography (UHPLC) with UV or mass spectrometric detection [17–20], HPTLC [21], voltammetry [22, 23], and potentiometric sensors [24, 25] have been reported for the determination of DOX. While chromatographic methods such as HPLC and UHPLC provide high sensitivity, they often involve high operational costs, extensive solvent consumption, and the requirement for sophisticated instrumentation, which may not be feasible for routine benchtop quality control in resource‐limited settings. The novelty of the current work lies in the development of a versatile, multimechanistic matrix. By offering four distinct spectrophotometric pathways, this study provides analytical flexibility; a laboratory can select a method based on the specific requirements of the matrix or available chemical inventory, thus significantly reducing the environmental and economic footprint compared to traditional chromatographic assays [26].

This study aims to address these limitations by developing and validating four complementary spectrophotometric methods utilizing distinct reaction principles: (i) a redox‐derived Turnbull’s blue system formed through Fe(III)/Fe(II) reduction, (ii) diazo‐coupling with Fast Red B (2‐methoxy‐4‐nitrobenzene‐diazonium‐1,5‐naphthalene disulfonate) in alkaline conditions to generate an azo chromogen (Figure 1b), (iii) oxidative transformation using NBS (1‐bromo‐2,5‐pyrolidinedione: succinbromimide) in an acidic medium (Figure 1c), and (iv) selective ion‐pair formation with anionic dyes followed by chloroform extraction using BTB (4,4′‐(3H‐2,1‐benzoxathiol‐3‐ylidene)bis[2‐bromo‐3‐methyl‐6‐(1‐methylethyl)‐,S,S‐dioxide; thymol,6,6′‐(3H‐2,1‐benzoxathiol‐3‐ylidene)bis[2‐bromo‐,S,S‐dioxide(8CI); 3,3′‐dibromothymolsulfonphthalein) (Figure 1d). To build on this, this research is innovative due to its comparative precision by validating and benchmarking four distinct paths according to ICH guidelines; we offer a diverse analytical method. This facilitates the strategic selection of a method according to particular sensitivity requirements, matrix complexity, or available laboratory equipment, thus setting a new standard for the accessible and sustainable quality control of DOX.

## 2. Experimental

### 2.1. Chemicals and Reagents

All reagents used were of analytical grade, and the solvents were of spectroscopic purity. Throughout the investigation, double‐distilled water was employed. A certified reference standard of DOX (99.9% purity) was provided by El‐Nile Company for Pharmaceuticals and Chemical Industries (Amiriya, Egypt). Pharmaceutical formulations (Doxymycin capsules, Batch No. 426007), labeled to contain 100 mg of DOX per capsule, was a gift from El‐Nile Company for Pharmaceuticals and Chemical Industries (Amiriya, Egypt).

Ferric chloride (FeCl_3_) and potassium ferricyanide (K_3_[Fe (CN)_6_]) were obtained from El‐Nasr Pharmaceutical Chemicals Co. in Egypt. Daily, 0.2% (w/v) aqueous solutions of these compounds were prepared. A 10 M aqueous solution was prepared, utilizing sulfuric acid (H_2_SO_4_) obtained from Merck, Germany. We prepared a 0.2% (w/v) solution of Fast Red B salt (Sigma‐Aldrich, Germany) in methanol (Sigma‐Aldrich) and a 2.5 × 10^−3^ M solution; these were filtered and protected from light. Sodium hydroxide (NaOH) (0.4% w/v) El‐Nasr Company) was used.

N‐Bromo succinimide (NBS) was purchased from Sigma‐Aldrich (Germany). A 1.0% (w/v) stock solution and a 1.0 × 10^−2^ M standard solution (0.18 g) were mixed in heated distilled water and stored them in the refrigerator to prevent decomposition. Glacial acetic acid (96%) and mineral acids (HCl and HNO_3_) were supplied by El‐Nasr Company (Egypt). Bromothymol Blue (BTB) was obtained from Win lab Laboratory Chemicals (U.K.). We prepared 0.1% (w/v) and 1.5 × 10^−3^ M solutions of the reagent in a small volume of ethanol (El‐Nasr Company) and diluted to the final volume with distilled water. Potassium acid phthalate buffer solution (pH 1.4–4.0) was purchased from El‐Nasr Company (Egypt), which was prepared by mixing 0.2 M potassium hydrogen phthalate with 0.2 M HCl, employing established methodologies for pharmaceutical preparation.

### 2.2. Apparatus

A dual‐beam UV–visible spectrophotometer (Shimadzu 1800) was implemented for spectrophotometric measurements using a quartz cell of a 1.0 cm path length. The software of Shimadzu UV Probe 2.32 was used for data sampling. A single scanning mode with a slit width of 1.0 nm and a sampling interval of 0.2 nm was utilized during the study.

### 2.3. Preparation of Standard Solutions

*Method A*: A stock solution of DOX (0.50 mg mL^−1^) was prepared by dissolving 50 mg of the compound in 50 mL of distilled water and diluting with an equal volume of distilled water to a total volume of 100 mL. A working standard solution (0.020 mg mL^−1^) was obtained by appropriate dilution of the stock solution with distilled water.

*Method B*: A stock solution of DOX (1.0 mg mL^−1^) was prepared by dissolving 50 mg of the compound in water and diluting to a final volume of 50 mL. A working standard solution (0.50 mg mL^−1^) was prepared by dilution of the stock solution with water. Additionally, a working solution (1.288 mg mL^−1^) was prepared by dissolving 0.128 g of the compound in distilled water and diluting to 100 mL.

*Method C*: A stock solution of DOX (1.0 mg mL^−1^) was prepared by dissolving 50 mg of the compound in water and diluting to 50 mL. A working solution (5.15 mg mL^−1^) was prepared by dissolving 0.515 g of the compound in distilled water and diluting to a total volume of 100 mL.

*Method D*: A stock solution of DOX (1.0 mg mL^−1^) was prepared by dissolving 50 mg of the compound in water and diluting to 50 mL. A working solution (0.50 mg mL^−1^) was obtained by diluting the stock solution with distilled water. Additionally, a working solution (0.773 mg mL^−1^) was prepared by dissolving 0.077 g of the compound in distilled water and diluting to a final volume of 100 mL.

### 2.4. Procedures

#### 2.4.1. Spectral Characteristics

**FIGURE 2:**
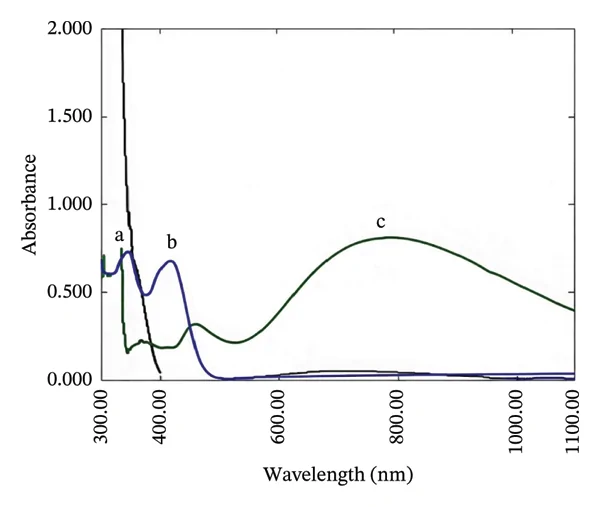
Absorption spectra of (a) 15 μg mL^−1^ of doxycycline hyclate in water, (b) FeCl_3_/ferricyanide reagent, and (c) 2 μg mL^−1^ of reaction product.

**FIGURE 3:**
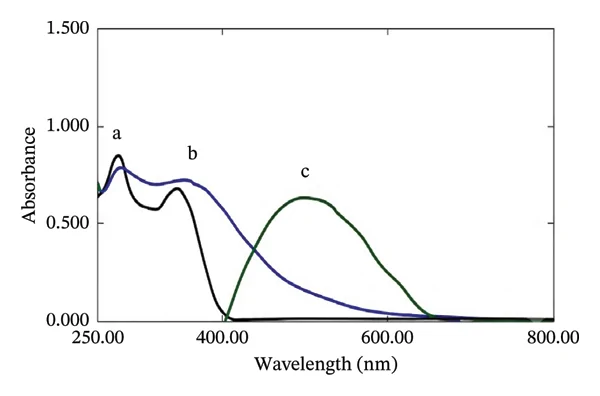
Absorption spectra of (a) 15 μg mL^−1^ of doxycycline hyclate in water, (b) Fast Red B reagent, and (c) 30 μg mL^−1^ of reaction product.

**FIGURE 4:**
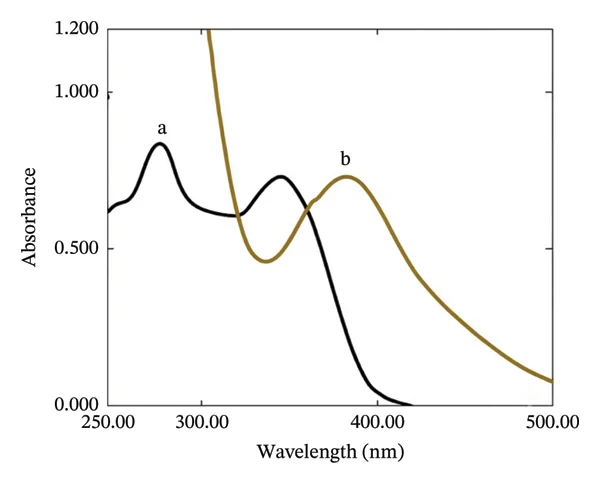
Absorption spectra of (a) 15 μg mL^−1^ of doxycycline hyclate in water, (b) 100 μg mL^−1^ of NBS–DOX reaction product.

**FIGURE 5:**
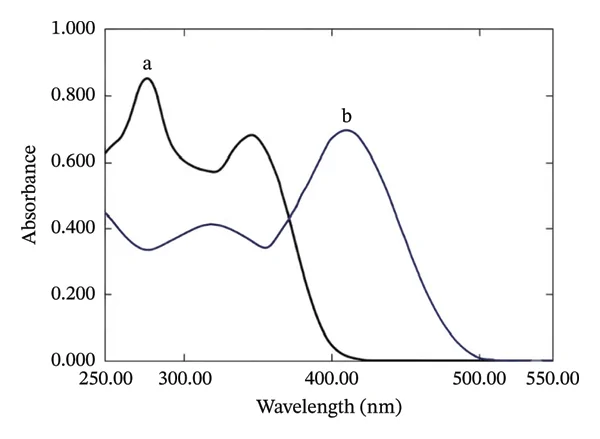
Absorption spectra of (a) 15 μg mL^−1^ of doxycycline hyclate in water, (b) 25 μg mL^−1^ of BTB–DOX reaction product.

Four derivatization methods were evaluated for the colorimetric quantification of DOX. In Method A, iron (III) is reduced to iron (II), which then reacts with ferricyanide to form a Turnbull’s blue complex, with absorbance recorded at 783.5 nm (Figure 2). In Method B, the reaction of DOX with the diazonium salt Fast Red B in an alkaline solution yielded a red azo dye that exhibits absorption at 498 nm (Figure 3). In Method C (NBS system), the interaction of DOX (100 μg/mL) with NBS in 96% acetic acid yielded a yellow product with a significant absorbance peak at 383.5 nm (Figure 4). In Method D (ion‐pair formation with BTB), upon interaction with BTB at pH 3, DOX produced a vibrant yellow ion‐pair complex. The chloroform extract of this chemical exhibited an absorbance maximum at 409.5 nm (Figure 5).

#### 2.4.2. Optimization of Reaction Conditions

##### 2.4.2.1. Method A (Iron (III)/Ferricyanide System)

*Effect of* FeCl_3_ *volume*: The influence of iron (III) concentration was evaluated by adding different volumes (0.5–4.0 mL) of 0.2% FeCl_3_ into 1.0 mL of DOX working standard solution (0.020 mg mL^−1^), followed by the addition of 2.5 mL of 0.2% potassium ferricyanide.

*Effect of potassium ferricyanide volume:* The concentration of the second reagent was optimized by examining various volumes (0.5–4.0 mL) of 0.2% potassium ferricyanide, while keeping the volume of Fecl_3_ constant.

*Effect of* H_2_SO_4_ *volume:* The acidity of the reaction medium was optimized by introducing different volumes (0.25–2.0 mL) of 10 M H_2_SO_4_.

*Effect of time*: The reaction mixtures were allowed to remain for different intervals, extending to 60 min, to establish the optimum reaction time.

##### 2.4.2.2. Method B (Fast Red B System)

*Effect of Fast Red B volume:* The effect of the coupling reagent was investigated using 0.6 mL of DOX working standard solution (0.50 mg mL^−1^) by adding varying volumes (1.0–8.0 mL) of 0.2% Fast Red B.

*Effect of NaOH volume*: The alkalinity of the reaction medium was optimized by testing different volumes (0.25–2.0 mL) of 0.4% NaOH solution.

*Effect of reaction time*: The stability and optimal development time of the azo dye were assessed by measuring the absorbance at 498 nm at different time intervals up to 60 min.

##### 2.4.2.3. Method C (NBS System)

*Effect of NBS reagent volume*: The reaction conditions were established using 1.0 mL of DOX standard solution (1.0 mg mL^−1^). The volume of the oxidizing agent was optimized by adding varying volumes (0.5–4.0 mL) of 1% NBS reagent.

*Effect of acetic acid volume*: The influence of the reaction medium was examined by adding varying volumes (1.0–6.5 mL) of 96% acetic acid.

*Effect of reaction time*: The reaction mixtures were allowed to stand for different time intervals up to 60 min to determine the optimum reaction time.

##### 2.4.2.4. Method D (Ion‐Pair Formation With BTB)

*Effect of pH:* The extraction parameters were optimized using 1.25 mL of DOX standard solution (0.50 mg mL^−1^). The influence of pH was investigated using 5.0 mL of phthalate buffer over the pH range of 1.4–4.0.

*Effect of buffer volume*: The volume of phthalate buffer (pH 3) was subsequently optimized over the range of 1.0–8.0 mL.

*Effect of dye volume*: The concentration of the counter‐ion was investigated by adding varying volumes (1.0–9.0 mL) of 0.1% BTB solution.

*Effect of standing time:* The influence of the standing time before extraction was evaluated by allowing the aqueous phase to stand for varying intervals up to 60 min.

*Effect of shaking time*: The extraction efficiency was assessed by varying the shaking time between 0.5 and 4.0 min.

### 2.5. Determination of Reaction Stoichiometry

The stoichiometry of the reactions was analyzed using the molar ratio method for methods B, C, and D [27].

In Method B, 1.0 mL of a 2.5 × 10^−3^ mol L^−1^ aqueous DOX solution was mixed with varying volumes (0.5–6.0 mL) of Fast Red B in methanol and 0.5 mL of 0.4% NaOH, followed by dilution and absorbance measurement at 498 nm. Method C involved the utilization of 1.0 mL of 1.0 × 10^−2^ mol L^−1^ DOX solution with different volumes (0.5–3.5 mL) of an NBS solution and 5.0 mL of 96% acetic acid, allowing a 40‐min reaction before measuring absorbance at 383.5 nm. Method D involved 1.0 mL of 1.5 × 10^−3^ mol L^−1^ DOX solution mixed with varying BTB solution volumes (0.5–6.0 mL) and adjusted with phthalate buffer (pH 3). The resulting ion‐pair complex was extracted with 10.0 mL of chloroform in a single extraction step, and the absorbance of the organic layer was measured at 409.5 nm.

### 2.6. Construction of Calibration Curves

Calibration curves were constructed by analyzing varying concentrations of DOX under the optimized conditions. In Method A, aliquots of working standard solution (0.020 mg mL^−1^) equivalent to 0.005–0.040 mg were treated with 2.5 mL each of 0.2% FeCl_3_ and 0.2% potassium ferricyanide; after 40 min, the reaction was acidified with 1.0 mL of 10 M H_2_SO_4_, diluted to 10 mL with water, and the absorbance was measured at 783.5 nm. For Method B, aliquots containing 0.05–0.60 mg DOX were mixed with 5.0 mL of 0.2% Fast Red B and 0.5 mL of 0.4% NaOH; following a 10 min standing period, the solutions were diluted to 10 mL and measured at 498 nm. In Method C, aliquots equivalent to 0.20–1.40 mg were reacted with 2.0 mL of 1% NBS and 5.0 mL of 96% acetic acid for 40 min, then diluted to 10 mL and measured at 383.5 nm. Finally, for Method D, aliquots containing 0.125–0.875 mg were mixed with 5.0 mL of phthalate buffer (pH 3) and 6.0 mL of 0.1% BTB in separating funnels; the aqueous phase was adjusted to 15 mL and extracted with 25 mL of chloroform, with the absorbance of the organic layer measured at 409.5 nm. In all cases, absorbance was plotted against the final drug concentration.

### 2.7. Application to Pharmaceutical Preparation

The contents of 10 doxymycin capsules, each containing 100 mg of DOX, were extracted, combined, and weighed. A precisely measured quantity of powder, corresponding to 50 mg of the drug, was transferred to a 100 mL volumetric flask, dissolved in distilled water, and diluted to the designated volume. The solution undergoes filtering to eliminate the excipients. Method A involves diluting an aliquot of the filtrate with water to achieve a final concentration of 0.020 mg mL^−1^. For procedures B and D, the filtrate was calibrated to a working concentration of 0.50 mg/mL. In procedure C, the filtrate was utilized directly or diluted to get a concentration of 1.0 mg/mL. The produced solutions were evaluated, and the concentration was measured using appropriate regression models. To ascertain the validity of the suggested processes and to mitigate potential interference from pharmaceutical excipients, the standard addition method was utilized to augment the preanalyzed capsule solutions with known quantities of purified DOX.

### 2.8. Method Validation

The method validation was executed to comply with ICH guidelines, including the assessment of linearity, range, accuracy, precision, limits of detection (LOD), limits of quantitation (LOQ), and robustness [28].

#### 2.8.1. Linearity and Range

Calibration curves were constructed by graphing the absorbance of DOX standard solutions in relation to their corresponding concentrations. The solutions were produced in triplicate, with concentrations ranging from 0.5 to 4 μg/mL (Method A), 5–60 μg/mL (Method B), 20–140 μg/mL (Method C), and 5–35 μg/mL (Method D), and the correlation coefficients were calculated.

#### 2.8.2. Accuracy

The methods’ accuracy was assessed by analyzing purified DOX samples at various concentration levels within the desired linear range. The percentage recovery (%R) was calculated using the regression equations.

#### 2.8.3. Precision

The precision was evaluated by intraday (repeatability) and interday (intermediate precision) assessments. Three concentrations of DOX were evaluated in triplicate on one day and on three successive days. Relative standard deviation (RSD%) is calculated to validate the accuracy of the approach.

#### 2.8.4. LOD and LOQ

The LOD and LOQ were determined using the equations LOD = 3.3 × *σ*/*S* and LOQ = 10 × *σ*/*S*, where *σ* represents the standard deviation of the response and S is the slope of the calibration curve.

#### 2.8.5. Robustness

We evaluated the robustness by implementing minor deliberate to essential experimental parameters, including varying the volume of the reagent and pH. The impact of these changes on the analytical response was monitored and expressed as relative standard deviation (RSD%).

## 3. Results and Discussion

### 3.1. Mechanistic Interpretation of the Derivatization Reactions

DOX exhibits a distinct absorption peak at 345 nm in an aqueous solution. Four spectrophotometric methods based on different reaction mechanisms were established to improve sensitivity and selectivity for its quantification. Each method depends on a specific chemical process that produces a colorful molecule with a unique absorption maximum.

Redox‐based method involves the reduction of Fe (III) to Fe (II) via the electron‐rich phenolic group of DOX and then interacting with ferricyanide to provide a mixed‐valence iron‐cyanide complex known as Turnbull’s blue. The strong absorption at 783.5 nm is due to intervalence charge‐transfer transitions in the Fe (II)–CN–Fe (III) lattice, as illustrated in Figure 2. The development of this extensively conjugated polymeric chromophore supports its remarkably high molar absorptivity and the enhanced sensitivity of the method (LOD 0.016 μg mL^−1^) in comparison to other approaches. For the coupling‐based method involving the conjugation of DOX with the diazonium salt Fast Red B, a broadly applicable technique was used for identifying phenolic and primary aromatic amine groups via the formation of colored azo dyes. In a slightly basic medium, Fast Red B undergoes electrophilic substitution at the activated aromatic or amino sites of the DOX framework, yielding a red azo chromophore with a maximal absorbance at 498 nm (Figure 3). This coupling technique produces a well‐conjugated dye that significantly shifts absorption into the visible region; the resulting extinction coefficient is lower than that of the iron‐ferricyanide complex in Method A. This reaction is very efficient in measuring DOX, and it produces a uniform and reliable signal that is suitable for regular drug analysis. The oxidative method utilizes N‐bromosuccinimide (NBS) as an electrophilic oxidant in a glacial acetic acid medium. NBS selectively targets the activated positions of DOX, yielding a yellow oxidation product with a maximum absorbance at 383.5 nm (Figure 4). The proposed mechanism involves an electrophilic oxidative transformation; given the structural features of DOX, the electrophilic bromine derived from NBS targets the electron‐dense regions of the hydronaphthacene scaffold. This reaction pathway is supported by the 1:1 stoichiometry determined via the molar ratio method (Figure S8b) and aligns with previously reported oxidative pathways for tetracycline analogs in acidic media [29, 30]. Glacial acetic acid serves as a non‐nucleophilic, protonating medium that stabilizes the oxidized species, preventing unwanted structural rearrangements. The observed sensitivity (LOD: 1.53 µ/mL) is consistent with the electronic characteristics of the resulting chromophore. A protonated amino group on DOX and an anionic sulfone phthalein dye, BTB, form a hydrophobic ion‐pair complex, which constitutes the foundation of the ion‐pair formation method at its optimal pH of 3.0; DOX predominantly exists in its protonated form, facilitating the removal of a proton from the phenolic group of the dye. This chemical reaction results in the formation of a quinoid structure and the opening of the BTB lactoid ring. The intense yellow coloration (*λ*_max_ = 409.5 nm) observed after extraction into chloroform is attributable to this interaction (Figure 5). Due to the liquid–liquid extraction method’s effective concentration of the analyte into the organic phase and providing of a clear optical background free from polar excipients, the approach exhibits a relatively high sensitivity (LOD 0.32 μg mL^−1^).

### 3.2. Optimization of Experimental Parameter

We systematically optimized the reaction parameters to get the best sensitivity and repeatability. We examined the amount of reagent, pH, reaction time, and the type of solvent all affecting the reaction. For redox‐based method, the generation of Turnbull’s blue depended on the concentration and acidity of the reagents. The maximum absorbance was reached with 2.5 mL, both FeCl_3_ and potassium ferricyanide (Figure S1). The reaction required a highly acidic environment, and 1.0 mL of 10 M H_2_SO_4_ produced the best color intensity (Figure S2). The redox process changed over time, with the absorbance increasing until it reached a stable maximum after 40 min (Figure S3). In coupling‐based method, the diazo‐coupling reaction was optimized. Fast Red B concentration had a significant effect on sensitivity, and 5.0 mL was found to be optimal, yielding maximum signal intensity (Figure S4a). The reaction necessitated a mildly alkaline media, with 0.5 mL of 0.4% NaOH producing the maximum absorbance; larger concentrations resulted in a reduction of signal intensity (Figure S4b). The coupling process was rapid, reaching maximal absorbance within 10 min and remaining stable for more than 1 hour (Figure S4c). For oxidative method, several acids were assessed, including HCl, H_2_SO_4_, HNO_3_, and acetic acid, with conclusions indicating that 96% acetic acid yielded the most stable, clear yellow chromogen. Figure S5a and Figure S5b indicate that the combination of 2.0 mL of 1% NBS and 5.0 mL of 96% acetic acid yielded the most favorable outcomes, and 40 min of incubation was adequate to complete the oxidative process (Figure S5c). Ion‐pair method exhibited considerable sensitivity to the pH of the aqueous phase. The ion‐pair complex was optimally extracted at pH 3.0 using 5.0 mL of phthalate buffer (Figure S6a and Figure S6b). The optimal dye concentration was achieved with 6.0 mL of BTB (Figure S6c). The complex was immediately produced at ambient temperature and remained stable for 1 hour (Figure S7a). A shaking time of 1 min was adequate for quantitative extraction (Figure S7b).

### 3.3. Stoichiometry and Reaction Mechanism

Employing the molar ratio method, we determined the stoichiometry of the reaction in the coupling‐based method. This demonstrated a 1:2 (drug: reagent) ratio (Figure S8a), which validates the proposed azo‐coupling mechanism illustrated in Scheme S1. The oxidative method demonstrated that the molar ratio between DOX and NBS reagent was 1:1 (Figure S8b), consistent with the single‐site oxidation mechanism depicted in Scheme S2. Ion‐pair method demonstrated a 1:1 M ratio between DOX and BTB (Figure S8c), thereby confirming the formation of the ion‐pair complex depicted in Scheme S3. In the redox‐based method, the stoichiometry reflects the reduction of Fe(III) complexed with ferricyanide ions upon reaction with DOX.

### 3.4. Validation Method

#### 3.4.1. Linearity and Range

The linearity of the proposed approaches was evaluated by examining a series of DOX solutions under optimal conditions. Calibration curves have been developed by plotting absorbance against the matching drug concentrations. The methodologies yielded linear responses within the subsequent concentration ranges: 0.5–4 μg mL^−1^ (Method A), 5–60 μg mL^−1^ (Method B), 20–140 μg mL^−1^ (Method C), and 5–35 μg mL^−1^ (Method D) (Figure S9). The high correlation coefficients (*r* ^2^ = 0.9994–0.9998) indicate excellent linearity. The regression parameters are listed in Table 1.

**TABLE 1:** Regression parameters for the determination of doxycycline hyclate by the proposed methods.

Parameter	FeCl_3_ and ferricyanide method	Fast Red B method	NBS method	BTB method
*λ*_max_ (nm)	783.5	498	383.5	409.5

Linearity range (μg mL^−1^)	0.5–4	5–60	20–140	5–35

Slope	0.3326	0.0203	0.0074	0.0276

Intercept	0.0103	0.041	0.0046	0.0023

SD of residual	5.6E − 04	1.4E − 04	4.1E − 04	1.1E − 04

Correlation coefficient (*r* ^2^)	0.9995	0.9999	0.9993	0.9998

Accuracy (R%)	100.91	99.70	99.83	99.81

LOD (μg mL^−1^)	0.016	1.09	1.53	0.32

LOQ (μg mL^−1^)	0.05	3.32	4.64	0.97
Precision (RSD%) Intraday precision^∗^ Interday precision^∗∗^	0.70 0.99	0.77 0.97	0.83 1.05	1.35 1.29

Robustness (RSD%)	0.64	1.60	1.06	1.8

^∗^The intraday (*n* = 3), average of three different concentrations repeated three times within the same day.

^∗∗^The interday (*n* = 3), average of three different concentrations repeated three times in three successive days.

#### 3.4.2. Accuracy

The accuracy was assessed using the analysis of pure DOX samples at various concentration levels within the linear range. The calculated concentrations were very close to the standard values. This proves that all the methods used were accurate as shown in Table 1.

#### 3.4.3. Precision

Precision was assessed utilizing repeatability (intraday) and intermediate precision (interday) studies. For each method, three distinct concentrations of DOX were analyzed: 1, 1.5, and 2 μg mL^−1^ for Method A; 10, 30, and 60 μg mL^−1^ for Method B; 40, 80, and 120 μg mL^−1^ for Method C; and 10, 20, and 30 μg mL^−1^ for Method D. Intraday precision was determined by analyzing each concentration in triplicate on the same day, while interday precision was assessed by repeating the analysis over three consecutive days. According to Table 1, the relative standard deviation (RSD%) values were within acceptable limit, indicating the precision of the proposed method.

#### 3.4.4. LOD and LOQ

The LOD and LOQ were calculated using this formula

(1) LOD=3.3×σS,LOQ=10×σS,

where *σ* is the standard deviation from the origin and S represents the slope. The LOD values for Methods A, B, C, and D were 0.016, 1.09, 1.53, and 0.32 μg mL^−1^, respectively, whereas the LOQ values were 0.05, 3.32, 4.64, and 0.97 μg mL^−1^, respectively (Table 1).

#### 3.4.5. Robustness

To evaluate the robustness of the proposed approaches, minor, deliberate modifications were implemented to the optimal experimental parameters. The volumes of FeCl_3_ and potassium ferricyanide utilized in Method A varied between 2 and 3 mL. In Method B, the concentration of 0.4% NaOH was modified slightly. The robustness of Methods C and D was assessed by varying the amount of the NBS reagent (1.8–2.2 mL) and the pH of the phthalate buffer (2.2–3.2), respectively. We monitored the impact of these modifications on the analytical response and documented it as a relative standard deviation (RSD%) (Table 1).

### 3.5. Application to Pharmaceutical Formulations

The procedures were effectively utilized for the determination of DOX in doxymycin capsules. The assay results closely aligned with the label specifications, with average recoveries ranging from 99.1% to 101.8%. The standard addition method further confirmed the accuracy and lack of matrix effects (Table 2).

**TABLE 2:** Application of standard addition technique for the determination of doxycycline hyclate by the proposed methods.

Preparation	FeCl_3_ and ferricyanide method				Fast Red B method				NBS method				BTB method			
	Mean ± SD%	Claimed taken μgmL^−1^	Pure added μgmL^−1^	Recovery % of added	Mean ± SD%	Claimed taken μgmL^−1^	Pure added μgmL^−1^	Recovery % of added	Mean ± SD%	Claimed taken μgmL^−1^	Pure added μgmL^−1^	Recovery % of added	Mean ± SD%	Claimed taken μgmL^−1^	Pure added μgmL^−1^	Recovery % of added
Doxymycin capsules.	100.3	0.5	0.5	100.0	99.1	20	10	100.3	99.2	50	20	99.6	100.9	10	10	102.0
	±1.04	0.5	1	100.3	±1.57	20	20	99.1	±1.43	50	30	97.7	±1.53	10	15	101.2
		0.5	1.5	99.5		20	30	97.9		50	50	99.6		10	20	101.0
		0.5	2	99.4		20	40	99.9		50	70	101.0		10	25	103.0

Mean% ± SD				99.8				99.3				99.5				101.8
				±0.42				±1.06				±1.37				±0.91

### 3.6. Comparative Analytical Performance and Best Results

**FIGURE 6:**
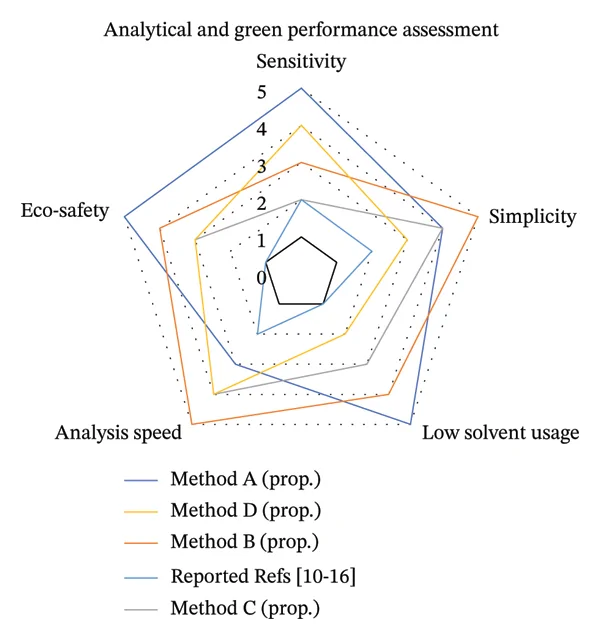
Radar chart comparing the analytical performance and green metrics of the four proposed methods (A–D) against the average performance of literature‐reported spectrophotometric methods ([10–16]).

A comparative assessment of the four methods indicates that Method A offers the greatest analytical sensitivity, establishing it as the optimal option for trace analysis or low‐dose formulations. This is primarily attributable to the “chemical amplification” effect associated with the formation of Turnbull’s blue, whereby the redox cascade yields a highly efficient polymeric product. Conversely, Method B provides the fastest turnover time (10 min), which is beneficial for high‐throughput routine quality assurance. Method D offers enhanced selectivity via solvent extraction, whereas Method C functions as a reliable alternative for higher concentration ranges. All methods exhibited excellent linearity (*r* ^2^ > 0.999) and were validated in accordance with ICH guidelines. A statistical comparison with the reference spectrophotometric method [16], utilizing Student′s *t*‐test and F‐test, demonstrated no significant differences in accuracy or precision at the 95% confidence level (Table 3). To highlight the novelty and significance of the current work, a critical comparison was performed against previously reported methods, as summarized in Table 4. The proposed methods demonstrate distinct advantages in terms of analytical sensitivity and sustainability. Notably, Method A exhibits the lowest limit of detection (LOD = 0.016 μg/mL), significantly outperforming most reported spectrophotometric and flow‐injection assays. Furthermore, by employing primarily aqueous‐based reaction media, our approaches adhere to the principles of Green Analytical Chemistry (GAC), reducing the reliance on toxic organic solvents often required by chromatographic or traditional spectroscopic procedures. The integration of four asynchronous pathways provides laboratory flexibility, allowing for the strategic selection of a method based on matrix complexity and sensitivity requirements, as visually summarized in the green assessment radar chart (Figure 6).

**TABLE 3:** Quantitative assay of doxycycline hyclate in their medications with statistical evaluation of the obtained results with a reported spectrophotometric one.

Parameters	FeCl method	Fast Red B method	NBS method	BTB method	Reported method^b^ ^(16)^
Mean %	100.3	99.1	99.2	100.9	99.3
SD^a^	1.04	1.57	1.43	1.53	1.98
Variance^a^	1.08	2.46	2.04	2.34	3.92
*N*	5	5	5	5	5
Student’s *t*‐test^c^ (2.306)	0.96	0.25	0.14	1.40	
F‐test^c^ (6.39)	3.63	1.59	1.92	1.68	

^a^Average of five determinations.

^b^UV spectrophotometric measurement yellow chromogen of doxycycline hyclate in 0.1 M NaOH at 375 nm.

^c^The values in parentheses represent the tabulated two‐tailed values at *p* = 0.05*.*

**TABLE 4:** Comparative analysis of the proposed spectrophotometric methods versus reported analytical techniques for doxycycline hyclate determination.

Detection technique	Linear range (μg/mL)	LOD (μg/mL)	Reference
Chemometric‐assisted ultraviolet spectrophotometry	5–25	—	[10]
Ultraviolet spectrophotometry	5–50	0.53	[11]
Ultraviolet spectrophotometry	2–20	1.41	[12]
Colorimetric spectrophotometry	0.5–35	0.24	[13]
Flow injection analysis/batch spectrophotometry	1.0–80	0.90	[14]
Flow injection analysis spectrophotometry	1.0–180	0.46	[15]
Ultraviolet–visible spectrophotometry	1.5–100	—	[16]
Redox spectrophotometric method (Turnbull’s blue reaction)	0.5–4	0.016	Method A (proposed)
Diazo‐coupling spectrophotometric method	5–60	01.09	Method B (proposed)
N‐Bromosuccinimide‐mediated oxidative spectrophotometric method	20–140	1.53	Method C (proposed)
Ion‐pair complex formation spectrophotometric method (bromothymol blue)	5–35	0.32	Method D (proposed)

### 3.7. Assessment Methodology of Analytical and Greenness Performances

To critically evaluate the environmental profile and operational practicality of the proposed spectrophotometric methods (A–D), a comprehensive greenness assessment was conducted. The evaluation was structured around key metrics aligned with the principles of GAC, including reagent toxicity, solvent consumption, energy requirements, generation of hazardous waste, and analysis time. Each parameter was scored on a standardized scale based on the nature of the reagents used (e.g., preference for aqueous media over toxic organic solvents), the simplicity of the sample preparation steps, and the overall energy footprint. As illustrated in Figure 6, the assessment is visualized using a radar chart where the concentric polygon lines represent the scoring scale from 0 (lowest performance) to 5 (highest performance), while the colored bounding lines represent the overall performance profile of each method across the five evaluated parameters (sensitivity, simplicity, low solvent usage, analysis speed, and eco‐safety). The resulting scores for methods A through D demonstrate that the proposed methods offer a significantly reduced environmental impact and higher operational simplicity compared to conventional chromatographic techniques, without compromising analytical reliability.

## 4. Conclusion

This study intentionally disrupts that resource‐intensive paradigm. Rather than delegating the separation to an expensive chromatography column, we compelled the polyfunctional structure of DOX to determine its own analytical parameters. By methodically delineating particular reactive locations on the drug’s fundamental scaffold, we developed four asynchronous, markedly separate chemical pathways that accomplish complete resolution without physical separation. It functioned exceptionally effectively. We effectively designed a system in which the structural mutability of the analyte serves as a substitute for the mobile phase. The validation data demonstrate that this structural exploitation is not only theoretical. Utilizing the DOX scaffold via various mechanisms polymeric redox complexation, basic diazo coupling, a highly climate‐sensitive kinetic oxidation, and lipophilic ion‐pairing resulted in a significant reduction of detection limits, achieving a minimal LOD of 0.16 μg mL^−1^. The test profile remains consistent. According to stringent ICH standards, the measurements indicated no susceptibility to systemic drift in terms of linearity, precision, or recovery configurations. Significantly, this is where benchtop approaches typically falter commercial capsule matrices produced no excipient‐induced signal attenuation or background noise during direct, raw‐sample derivatization. This achievement in concrete operations dispels the notion that extensive chromatographic infrastructure is essential for standard pharmaceutical quality control. The intrinsic structural plasticity of an analyte offers a direct, very sensitive spectroscopic readout, rendering the need of cumbersome, solvent‐intensive instruments a redundant logistical endeavor.

## Author Contributions

Mona A. Abdel Rahman: conceptualization of the work, formal analysis, investigation, visualization of the data, methodology, validation, and writing–review and editing. Reem H. Obaydo: validation, methodology, and writing–review and editing.

## Funding

This research did not receive any specific grant from public, commercial, or not‐for‐profit funding agencies.

## Conflicts of Interest

The authors declare no conflicts of interest.

## Supporting Information

Additional supporting information can be found online in the Supporting Information section.

## Supporting information


**Supporting Information** Figure S1. Optimization of reagent volumes for effect of (a) 0.2% (w/v) FeCl_3_ and (b) 0.2% (w/v) potassium ferricyanide on the absorbance of the DOX–Turnbull’s blue system (2 μg mL^−1^) at 783.5; Figure S2. Effect of volume of 10 M H_2_SO_4_ on absorbance of reaction product of DOX (2 μg mL^−1^) at 783.5 nm; Figure S3. Effect of reaction time on the absorbance intensity of the DOX–Turnbull’s blue complex (2 μg mL^−1^) at 783.5 nm; Figure S4. Influence of (a) 0.2% (w/v) Fast Red B volume, (b) 0.4% (w/v) NaOH volume, and (c) reaction time on the absorbance of the DOX–azo‐dye chromogen (30 μg mL^−1^) at 498 nm; Figure S5. Influence of (a) 1.0% (w/v) NBS volume and (b) 96% (v/v) acetic acid volume on the absorbance of the resulting DOX oxidation product (100 μg mL^−1^) at 383.5 nm; Figure S6. Influence of (a) phthalate buffer pH, (b) buffer volume, and (c) 0.1% (w/v) BTB volume on the absorbance of the DOX–BTB complex (25 μg mL^−1^) at 409.5 nm; Figure S7. Effect of (a) reaction time and (b) shaking time on the absorbance of the DOX–BTB ion‐pair complex (25 μg mL^−1^) at 409.5 nm; Figure S8. Stoichiometry of the reaction of DOX by molar ratio method with a) 2.5 × 10^−3^ M Fast Red B, b) 1 × 10^−2^ M NBS, and c) 1.5 × 10^−3^ M BTB; Figure S9. Linear regression plots of absorbance versus concentration for (a) Method A (Turnbull’s blue, 783.5 nm), (b) Method B (Fast Red B, 498 nm), (c) Method C (NBS, 383.5 nm), and (d) Method D (BTB, 409.5 nm); Scheme S1. Schematic representation of the electrophilic diazo‐coupling between the phenolic moiety of DOX and the Fast Red B; Scheme S2. Schematic mechanism illustrating the NBS‐mediated oxidation of the DOX polyhydroxylated system; Scheme S3. Schematic representation of the electrostatic interaction between the protonated dimethylamino group of DOX and BTB at pH 3.0.

## Data Availability

The data that support the findings of this study are available from the corresponding author upon reasonable request.

## References

[1] Ali T. , Mohamed G. , El-Sonbati A. , Diab M. , and Elkfass A. , A Potentiometric Sensor for Determination of Doxycycline Hydrochloride in Pharmaceutical Preparation and Biological Fluids, Journal of Electrochemical Science and Technology. (2018) 54, no. 12, 1081–1095, 10.1134/s1023193518120029.

[2] Mei C. , Li L. , Zhang J. et al., Ion-Induced Enhanced Fluorescence Colorimetric Hydrogel Sensor for Visual Quantization of Doxycycline, Sensors and Actuators, B: Chemical. (2023) 380, 10.1016/j.snb.2023.133359.

[3] Sloan B. and Scheinfeld N. , The Use and Safety of Doxycycline Hyclate and Other Second-Generation Tetracyclines, Expert Opinion on Drug Safety. (2008) 7, no. 5, 571–577, 10.1517/14740338.7.5.571. 18759709

[4] Monk E. , Shalita A. , and Siegel D. M. , Clinical Applications of Non-Antimicrobial Tetracyclines in Dermatology, Pharmacological Research. (2011) 63, no. 2, 130–145, 10.1016/j.phrs.2010.10.007. 20937386

[5] Shang S. , Du F. , Feng H. , and Wu Y. , Comparisons of Efficacy and Safety of Different Doses of Doxycycline for the Treatment of Moderate‐To‐Severe Acne Vulgaris: A Systematic Review and Network Meta‐Analysis, Dermatologic Therapy. (2025) 2025, no. 1, 10.1155/dth/1713121.

[6] Abu-Awwad A. N. , Muti H. Y. , Khater D. F. , Khalaf A. M. , and Arafat T. A. , Simultaneous Determination of Ciprofloxacin, Enrofloxacin, Doxycycline, and Chloramphenicol Residues in Poultry, Red Meat, and Fish by High-Performance Liquid Chromatography Ultraviolet Method, Open Veterinary Journal. (2025) 15, no. 9, 10.5455/ovj.2025.v15.i9.47. PMC12587809 41200290

[7] Govind V. , Narendra Babu R. , Appa Rao V. , Sriram P. , and Senthil Kumar T. , Determination of Sulfadoxine Residues in Poultry Meat by Liquid Chromatography and Tandem Mass Spectrometry, Journal of Food Science and Technology. (2018) 55, 2110–2118.

[8] Wormser G. P. , Doxycycline for Prevention of Spirochetal Infections: Status Report, Clinical Infectious Diseases. (2020) 71, no. 8, 2014–2017, 10.1093/cid/ciaa240. 32157268

[9] Farkas N.-I. , Marincaş L. , Barbu-Tudoran L. , Barabás R. , and Turdean G. L. , Investigation of the Real-Time Release of Doxycycline From PLA-Based Nanofibers, Journal of Functional Biomaterials. (2023) 14, no. 6, 10.3390/jfb14060331. PMC10299625 37367295

[10] Eticha T. , Kahsay G. , Asefa F. et al., Chemometric‐Assisted Spectrophotometric Method for the Simultaneous Determination of Ciprofloxacin and Doxycycline Hyclate in Pharmaceutical Formulations, Journal of Analytical Methods in Chemistry. (2018) 2018, 9538435–5, 10.1155/2018/9538435. 30662790 PMC6312589

[11] Sahoo P. K. , Sahoo P. , Mohapatra J. , Panigrahi D. , Patra A. K. , and Mishra A. , Development and Validation of Stability Indicating Assay Method of Doxycycline Hyclate by Using UV-Spectrophotometer, Journal of Drug Delivery and Therapeutics. (2023) 13, no. 6, 45–52, 10.22270/jddt.v13i6.6087.

[12] Gholse Y. N. , Chaple D. R. , and Kasliwal R. H. , Development and Validation of Novel Analytical Simultaneous Estimation Based UV Spectrophotometric Method for Doxycycline and Levofloxacin Determination, Asian Journal of Pharmaceutical Analysis. (2022) 12, 5458–5478.

[13] Abdulsattar J. O. , Hadi H. , Richardson S. , Iles A. , and Pamme N. , Detection of Doxycycline Hyclate and Oxymetazoline Hydrochloride in Pharmaceutical Preparations via Spectrophotometry and Microfluidic Paper-Based Analytical Device (μPADs), Analytica Chimica Acta. (2020) 1136, 196–204, 10.1016/j.aca.2020.09.045. 33081945

[14] Abdulghani R. H. and Hassan R. F. , Development and Validation of Spectrophotometric Methods for the Quantitative Determination of Doxycycline Hyclate in Pure Form and Pharmaceutical Formulations Using Flow‐Injection and Batch Procedures: A Comparative Study, ChemistrySelect. (2023) 8, no. 13, 10.1002/slct.202300183.

[15] Mohammed T. and Hadi H. , Normal and Reverse Flow Injection-Spectrophotometric Determination of Doxycycline Hyclate in Bulk and Pharmaceutical Samples, Baghdad Science Journal. (2025) 22, no. 8, 2539–2549, 10.21123/2411-7986.5016.

[16] Ramesh P. J. , Basavaiah K. , Divya M. R. , Rajendraprasad N. , Vinay K. B. , and Revanasiddappa H. D. , Simple UV and Visible Spectrophotometric Methods for the Determination of Doxycycline Hyclate in Pharmaceuticals, Journal of Analytical Chemistry. (2011) 66, no. 5, 482–489, 10.1134/s1061934811050157.

[17] Usmani M. , Ahmed S. , Anwar Z. , Zafar F. , and Sheraz M. A. , A Smart and Eco-Friendly Analytical Quality by Design (AQbD) Approach for the Analysis of Doxycycline Hyclate in Pure, Pharmaceutical Dosage Forms, Stressed, and Biological Samples Using a Stability Indicating UHPLC Method, Spectrochimica Acta Part A: Molecular and Biomolecular Spectroscopy. (2025) 14, no. 2, 1–24, 10.1080/23080477.2025.2579256.

[18] Mahmoud O. A. , Omran A. A. , Gomaa H. A. , and Mohamed M. A. , Stability Indicating High‐Performance Liquid Chromatography Method for Determining Doxycycline Hyclate and Related Substances in Their Various Dosage Forms: Utilizing Six Sigma Tools, Uniformity Testing, and In‐Vitro Dissolution Approaches, Sep, Science Policy. (2024) 7, no. 2, 10.1002/sscp.202300173.

[19] Pippalla S. , Nekkalapudi A. R. , Jillellamudi S. B. , Reddy M. P. , and Kumar C. V. , A Stability‐Indicating, Reversed‐Phase HPLC Method for Quantification of Assay and Organic Impurities in Doxycycline Hyclate Bulk and Parenteral Dosage Forms, Biomedical Chromatography. (2023) 37, no. 6, 10.1002/bmc.5626. 36930874

[20] Magid N. and Hammood M. K. , New Methodology Colorimetric-SFI Analysis for the Determination of Doxycycline Hyclate in Pharmaceutical Preparations, Ibn Al-Haitham J. Pure, Applied Sciences. (2025) 38, 234–248.

[21] Kumssa L. , Layloff T. , Hymete A. , and Ashenef A. , High Performance Thin Layer Chromatography (HPTLC) Method Development and Validation for Determination of Doxycycline Hyclate in Capsule and Tablet Formulations, Journal of Planar Chromatography—Modern TLC. (2021) 115–122.

[22] Xu Z. , Jiang X. , Liu S. , and Yang M. , Sensitive and Selective Molecularly Imprinted Electrochemical Sensor Based on Multi-Walled Carbon Nanotubes for Doxycycline Hyclate Determination, Chinese Chemical Letters. (2020) 31, no. 1, 185–188, 10.1016/j.cclet.2019.04.026.

[23] Tiwari M. , Thorat R. , Popatkar B. , Borge V. , and Kadu A. , Voltammetric Determination of Doxycycline in Feedstock Using Modified Carbon Screen-Printed Electrode, Analytical Sciences. (2023) 39, no. 11, 1889–1899, 10.1007/s44211-023-00395-5. 37495926

[24] Asran A. M. , Mohamed M. A. , Abd El-Rahman M. K. , and Mousavi M. P. , Green Ecofriendly Electrochemical Sensing Platform for the Sensitive Determination of Doxycycline, Heliyon. (2023) 9. 10.1016/j.heliyon.2023.e15223 PMC10123195 37101647

[25] Sayed R. A. , Elmasry M. S. , Hassan W. S. , El-Mammli M. Y. , and Shalaby A. , The Use of Surface Plasmon Resonance Band of Green Silver Nanoparticles and Conductometry for Quantitative Determination of Minor Concentrations of Doxycycline Hyclate and Oxytetracycline HCl in Pure and Pharmaceutical Dosage Forms, International Journal of Nanomedicine. (2020) 16, no. 3, 203–220, 10.1504/ijnm.2020.108038.

[26] Al Zakri D. J. , Obaydo R. H. , and Sakur A. A. , New Spectral Resolution Techniques for Resolving and Determining the Components in Binary Fixed-Dose Combinations, Heliyon. (2019) 5, no. 10, 10.1016/j.heliyon.2019.e02637. PMC6820088 31687499

[27] Rose J. J. , Advanced Physico-Chemical Experiments: A Textbook of Practical Physical Chemistry and Calculations, 1964, Pitman.

[28] Ich Harmonised Tripartite Guideline , Pharmaceutical Development Q8(R2), Current Step 4 Version, International Conference on Harmonisation. (2009) .

[29] Halvatzis S. A. , Timotheou-Potamia M. M. , and Calokerinos A. C. , Continuous-Flow Chemiluminometric Determination of Tetracyclines in Pharmaceutical Preparations and Honey by Oxidation With N-Bromosuccinimide, Analyst. (1993) 118, no. 6, 633–637, 10.1039/an9931800633. 8342787

[30] Sheet R. I. and Mohammed S. A. , Indirect Spectrophotometric Method for the Quantitative Estimation of Tetracycline Hydrochloride in the Pharmaceutical Formulations, Annals of RSCB. (2023) 27, no. 1, 169–180.

