# Supplementary material for: Harnessing the Structural Variability of Doxycycline Hyclate: Mechanistic Insights and Comparative Assessment of Four Chemically Diverse Spectrophotometric Pathways
Source: J Anal Methods Chem. 2026 Aug 30;2026:2780011. doi: 10.1155/jamc/2780011 (PMC13526241; doi:10.1155/jamc/2780011)
Supplement: Supplementary file 1 [file JAMC-2026-2780011-s001.docx]

**Figures**

**Fig S1.** Optimization of reagent volumes for effect of (a) 0.2% (w/v) FeCl_3_ and (b) 0.2% (w/v) potassium ferricyanide on the absorbance of the DOX–Turnbull’s blue system (2 µg mL^-1^) at 783.5**.**

| Volume of 10 M H2SO4 (mL) | Absorbance |
| --- | --- |
| 0.25 | 0.334 |
| 0.5 | 0.541 |
| 0.75 | 0.688 |
| 1 | 0.704 |
| 1.25 | 0.7 |
| 1.5 | 0.699 |
| 1.75 | 0.7 |
| 2 | 0.692 |

**Fig S2.** Effect of volume of 10 M H_2_SO_4_ on absorbance of reaction product of doxycycline hyclate (2 µg mL^-1^) at 783.5 nm.

**Fig S3.** Effect of reaction time on the absorbance intensity of the DOX–Turnbull’s blue complex (2 µg mL^-1^) at 783.5 nm.

| Time (min) | Absorbance |
| --- | --- |
| 2 | 0.038 |
| 5 | 0.149 |
| 10 | 0.325 |
| 15 | 0.398 |
| 20 | 0.501 |
| 25 | 0.648 |
| 30 | 0.663 |
| 35 | 0.71 |
| 40 | 0.707 |
| 45 | 0.705 |
| 50 | 0.705 |
| 55 | 0.708 |
| 60 | 0.704 |

**Fig S4.** Influence of (a) 0.2% (w/v) Fast Red B volume, (b) 0.4% (w/v) NaOH volume, and (c) reaction time on the absorbance of the DOX–azo-dye chromogen (30 µg mL^-1^) at 498 nm.

**Fig S5.** Influence of (a) 1.0% (w/v) NBS volume, (b) 96% (v/v) acetic acid volume on the absorbance of the resulting DOX oxidation product (100 µg mL^-1^) at 383.5 nm. and (c) incubation time showing reaction completion at 40 min.

**Fig S6.** Influence of (a) phthalate buffer pH, (b) buffer volume, and (c) 0.1% (w/v) BTB volume on the absorbance of the DOX–BTB complex (25 µg mL^-1^) at 409.5 nm.

**Fig S7.** Effect of (a) reaction time and (b) shaking time on the absorbance of the DOX–BTB ion-pair complex (25 µg mL^-1^) at 409.5 nm.

**Fig S8.** Stoichiometry of the reaction of DOX by molar ratio method with a) 2.5x10^-3^ M fast red B, b) 1x10^-2^ M NBS and c) 1.5x10^-3^ M BTB.

**Scheme S1.** Schematic representation of the electrophilic diazo-coupling between the phenolic moiety of DOX and the Fast Red B

**Scheme S2.** Schematic mechanism illustrating the NBS-mediated oxidation of the DOX polyhydroxylated system

**Scheme S3.** Schematic representation of the electrostatic interaction between the protonated dimethylamino group of DOX and BTB at pH 3.0.

**Fig S9.** Linear regression plots of absorbance versus concentration for: (a) Method A (Turnbull’s blue, 783.5 nm), (b) Method B (Fast Red B, 498 nm), (c) Method C (NBS, 383.5 nm), and (d) Method D (BTB, 409.5 nm).
